# International medical graduates: a workforce at a crossroad?

**DOI:** 10.1177/01410768261421438

**Published:** 2026-02-24

**Authors:** Richard Antony Powell, Mo Al-Haddad, Vijay Nayar, Kam Bhui, Mala Rao

**Affiliations:** 1 Ethnicity and Health Unit, Department of Primary Care and Public Health, School of Public Health, Imperial College London, London, England; 2 Intensive Care Unit, Queen Elizabeth University Hospital, Glasgow, Scotland; 3 Anglia Ruskin University, Chelmsford, England; 4 Department of Psychiatry and Wadham College, University of Oxford, Oxford, England

Against a backdrop of ongoing resident doctors’ strike threats, in December 2025 Secretary of State for Health and Social Care, Wes Streeting, proposed to create 4000 medical specialty training places, with preference given to United Kingdom (UK) medical graduates (UKMGs), over the next 3 years.^1^ This poses a pivotal question for international medical graduates (IMGs): are they at a career crossroad?

The rationale underpinning the proposal was clear. Taxpayers currently spend £4 billion training medical doctors each year, but many leave the UK to work abroad or in the private sector. In 2024, 2627 UKMGs under the age of 40 left the General Medical Council’s (GMC) register, a rise of 17% from the year before.^2^ There is therefore, contended Mr Streeting, a need to ‘protect [taxpayers’] investment and give bright, hard-working medical graduates a path to become the next generation of NHS doctors’.^3^ The move to prioritise UKMGs over IMGs is also intended to reduce competition for training posts among resident doctors. The proposal would thereby ease the substantial bottleneck in career progression where an increasing number of doctors are applying for limited speciality training positions.^4^

As Mr Streeting noted: ‘Doctors asked me to deliver on jobs, especially unfair competition from overseas, and this comprehensive offer will deliver – providing resident doctors currently applying with more jobs, prioritising UK-trained doctors’.^1^ While the proposal was rejected in the subsequent doctors’ ballot, it confirmed a direction of travel signalled in the NHS *Ten-Year Plan*,^5^ that is, growing domestic training pipelines and reducing reliance on overseas recruitment over time.

There have been many comparable policy shifts aimed at reducing reliance on international workers, only to have them reversed when facing new political challenges, for example, Brexit. In this context, it is difficult to consider these policy decisions as long-standing or indeed remedial of the lack of strategic planning and coordination for medical training and provision of posts. There is a shortage of specialists and general practitioners,^6^ and yet many are unemployed or unable to progress in their training.

Irrespective of the sustainability of the policy redirection, IMGs, especially those currently in the UK, will face increased competition for, and reduced access to, specialty training opportunities and impaired career progression. In the balance between providing sufficient incentives to UKMGs to increase NHS recruitment and retention, and avoiding disincentivising, demoralising and alienating IMGs, the government appears to have prioritised the former.

Early signs of the impact of this prioritisation were recently outlined in the GMC’s 2025 *State of Medical Education and Practice* workforce report.^2^ In 2024, over 20,000 IMGs joined the medical register, matching the high volumes of previous years, filling positions in general practice, acute medicine, psychiatry and smaller district general hospitals.^2^ Forty-two percent of the licensed medical workforce are now IMGs.^2^ However, there has also been a steep rise in doctors leaving the NHS; 4880 IMGs relinquished their license in 2024, a 26% increase on the previous year.^2^ A likely explanation is that large numbers of IMGs (66% of the total) are recruited to locally employed doctor (LED) roles.^7^ These posts are often poorly defined, carry limited training progression potential and lack the pastoral support that can be found in formal training pathways.^2^ While LED roles are crucial for service delivery, they can unintentionally create a professional cul-de-sac for those IMGs aspiring to secure speciality training or long-term employment. Additionally, fewer newly licensed IMGs find employment quickly: in 2024, just 13% were connected to a ‘designated body’ (i.e. employer) within 6 months, down from 20% in 2023.^2^

The rising exit rate of IMGs also partly reflects their lived experiences of transition, dislocation and often isolation.^8^ Many IMGs report their early years in the UK as among the most challenging, particularly when navigating unfamiliar clinical norms, communication expectations, regulatory requirements and cultural differences.^9^ These pressures can be compounded by family separation, the financial burden of relocation, limited social networks^8^ and racist microaggressions.^10^ Together, they necessitate holistic welfare support encompassing pastoral care, cultural orientation, psychological well-being and career coaching, support that is often non-existent. Systems in crisis do not treat people well, so many arriving IMGs find themselves expected to meet unrealistic objectives, with insufficient support, and minimal time for personal development or career progression.

IMGs will be essential to the NHS for the foreseeable future.^5^ To ensure the ambitions of the *Ten-Year Plan* are met, patients are treated quickly and effectively, public health improves and the NHS does not collapse under the weight of ill-considered rapid policy initiatives, IMGs, especially those in the UK currently, must not be viewed as simply emergency gap-fillers but as long-term, valued partners in the NHS, as they have always been since its inception.^11^ The well-intentioned directional change proposed by Mr Streeting is not a long-term solution; workforce planning and training need to be taken far more seriously as the current system is wasteful, inefficient and unfair.

This requires a five-fold action: (1) career recruitment equity with a view to equality in outcomes – IMGs in the UK must have equity in competing for training posts and ensuring IMGs have routes into research, training and speciality-specific roles; (2) welfare as infrastructure – mandatory structured pastoral support for all new IMGs at Trust/Health Board level and embedding cultural safety training for supervisors and departmental leads; (3) retention through belonging – promoting well-being through equity and fostering strong social connections and community networks; (4) ethical leadership and management – needed to support IMGs and moderate the harsh impacts of failing health systems on doctors’ health and (5) workforce planning – using detailed modelling methods for a future workforce which can address population healthcare needs as well as reliably predict future IMG recruitment needs.

IMGs have chosen the path of contribution and commitment to the NHS since its birth,^11^ but they are now at a crossroad. Without urgent action, they will have no option but to change path and leave for other more welcoming and fairer healthcare systems. That would be a loss for the NHS, destabilising its medical workforce and compromising patient care.

## Declarations

**Ethics approval:** Not applicable.

**Guarantor:** MR.

**Contributorship:** RAP drafted the first version of the manuscript, with MAH, VN, KB and MR commenting and helping redraft it.

**Provenance:** Not commissioned; editorial review.

**Use of generative AI:** No generative AI was used during the preparation of this manuscript.
